# Intra-operative Guidelines for the Prevention of Uterine Niche Formation in Cesarean Sections: A Review

**DOI:** 10.7759/cureus.44521

**Published:** 2023-09-01

**Authors:** Sean Backer, Deepesh Khanna, Sonia Sadr, Ali Khatibi

**Affiliations:** 1 Osteopathic Medicine, Nova Southeastern University Dr. Kiran C. Patel College of Osteopathic Medicine, Tampa, USA; 2 Foundational Sciences, Nova Southeastern University Dr. Kiran C. Patel College of Osteopathic Medicine, Clearwater, USA; 3 Foundational Sciences, Nova Southeastern University Dr. Kiran C. Patel College of Osteopathic Medicine, Fort Lauderdale, USA; 4 Obstetrics and Gynaecology, Sahlgrenska University Hospital, Gothenburg, SWE

**Keywords:** cesarean sections, c-section, ectopic pregnancy, hysterotomy closures, intra-operative, mesenchymal stem cell (msc), placenta accreta, platelet-rich plasma (prp), uterine niche, uterine rupture

## Abstract

Formation of a uterine niche following a C-section can predispose the patient to future obstetric complications such as dehiscence, uterine rupture, ectopic pregnancy, and placenta accreta. The significant morbidity and mortality of these complications along with increasing C-section rates emphasizes the importance of prevention. However, there are no clear guidelines on intra-operative protocol to prevent postpartum niche formation. Besides surgical technique, the novel use of platelet-rich plasma (PRP) and mesenchymal stem cell (MSC) injections has demonstrated promising potential and may have applications in hysterotomy closures. The objective is to examine current research on optimal C-section procedures to prevent uterine niche formation and subsequent obstetric complications. A systematic review was conducted using PubMed and Google Scholar. Initial searches yielded 827 results. Inclusion criteria were human, animal, and in-vitro studies, peer-reviewed sources, and outcomes pertinent to the uterine niche. Exclusion criteria applied to articles with outcomes unrelated to myometrium and interventions outside of the intra-operative and immediate pre-/post-operative period. Based on the criteria, 41 articles were cited. Pathophysiology of uterine niche formation was associated with incisions through cervical tissue, adhesion formation, and poor approximation. Significant risk factors were low uterine incisions, advanced cervical dilatation, low station, non-closure of the peritoneum, and creation of a bladder flap. There was no consensus on uterine closure as it likely depends on surgical proficiency with the given technique, but a double-layered non-locking suture appears reliable to reduce niche severity. Recent trials indicate that intra-operative PRP/MSC injections may decrease niche incidence and severity, but more research is needed. If prevention or minimization of uterine niche is desired, the optimal C-section protocol should avoid low uterine incisions, choose uterine closure technique based on the surgeon’s proficiency (double-layered non-locking is reliable), and close the peritoneum, and myometrial injection of PRP/MSC may be a useful adjunct intervention pending further clinical evidence.

## Introduction and background

Cesarean sections are becoming increasingly common, but not without risk. Based on global data between 2010 and 2018, C-sections account for 21.1% of global live births, ranging from 5% in Sub-Saharan Africa to 42.8% in Latin America [1]. Furthermore, it is projected that global C-section rates may increase to 28.5% by the year 2030 [1]. However, the trend is not without risk, as C-sections are associated with numerous short- and long-term complications, specifically, the development of a uterine niche (isthmocele). Uterine niche is defined as a discontinuation of the myometrium and endometrium after hysterotomy as part of a C-section. Niche formation following a C-section is not uncommon, as ultrasonographic studies indicate an incidence between 56% and 84% (with sonohysterography) following an initial C-section, although ranges vary widely in different studies [2-4]. The high global rates of C-sections in combination with the frequent incidence of uterine niche formation yield a large population of women at risk for potentially severe complications in subsequent pregnancies. This anatomical alteration can predispose the patient to high-risk obstetric complications, such as pre-term delivery, miscarriage, uterine dehiscence, rupture, cesarean scar ectopic pregnancy, or placenta accreta in future pregnancies [2,5,6]. A meta-analysis demonstrated the incidence of miscarriage, pre-term delivery, and placenta accreta spectrum following cesarean niche formation as 19.1%, 10.3%, and 4.0%, respectively [5].

A uterine niche is usually detected as a triangular anechoic area or myometrial discontinuation by imaging modalities such as an initial transvaginal ultrasound (TVUS) or a more sensitive sonohysterogram (SHG), with 3D ultrasound, hysterosalpingography, and MRI being additional, albeit less utilized options [2,6,7]. Typically, the imaging is indicated due to gynecologic complaints related to an isthmocele, such as spotting, dysmenorrhea, pelvic pain, or dyspareunia [7]. The literature describes treatment approaches ranging from conservative treatment to laparoscopic and hysteroscopic resection and repair, depending on the severity of symptoms and future reproductive goals of the patient [6]. Although there is extensive research on different imaging modalities and treatment options for uterine niches, there is no clear consensus on optimal intra-operative guidelines during a C-section to prevent the future formation of a niche, especially pertaining to uterine closure. Furthermore, the potential benefits of platelet-rich plasma and mesenchymal stem cells have just recently been observed in infertility and reproductive medicine and may have potential as an adjunctive intervention to prevent uterine niche formation. As a result, the objective of this study is to systematically review the current literature and recent updates on intra-operative protocols during C-sections to prevent future uterine niche formation and subsequent complications. To accomplish this objective, the following research question is asked: what is the optimal protocol for C-section hysterotomy and closure, and can adjunct interventions provide additional benefit, in order to minimize future uterine niche formation and obstetric complications in subsequent pregnancies?

## Review

Materials and methods

**Figure 1:**
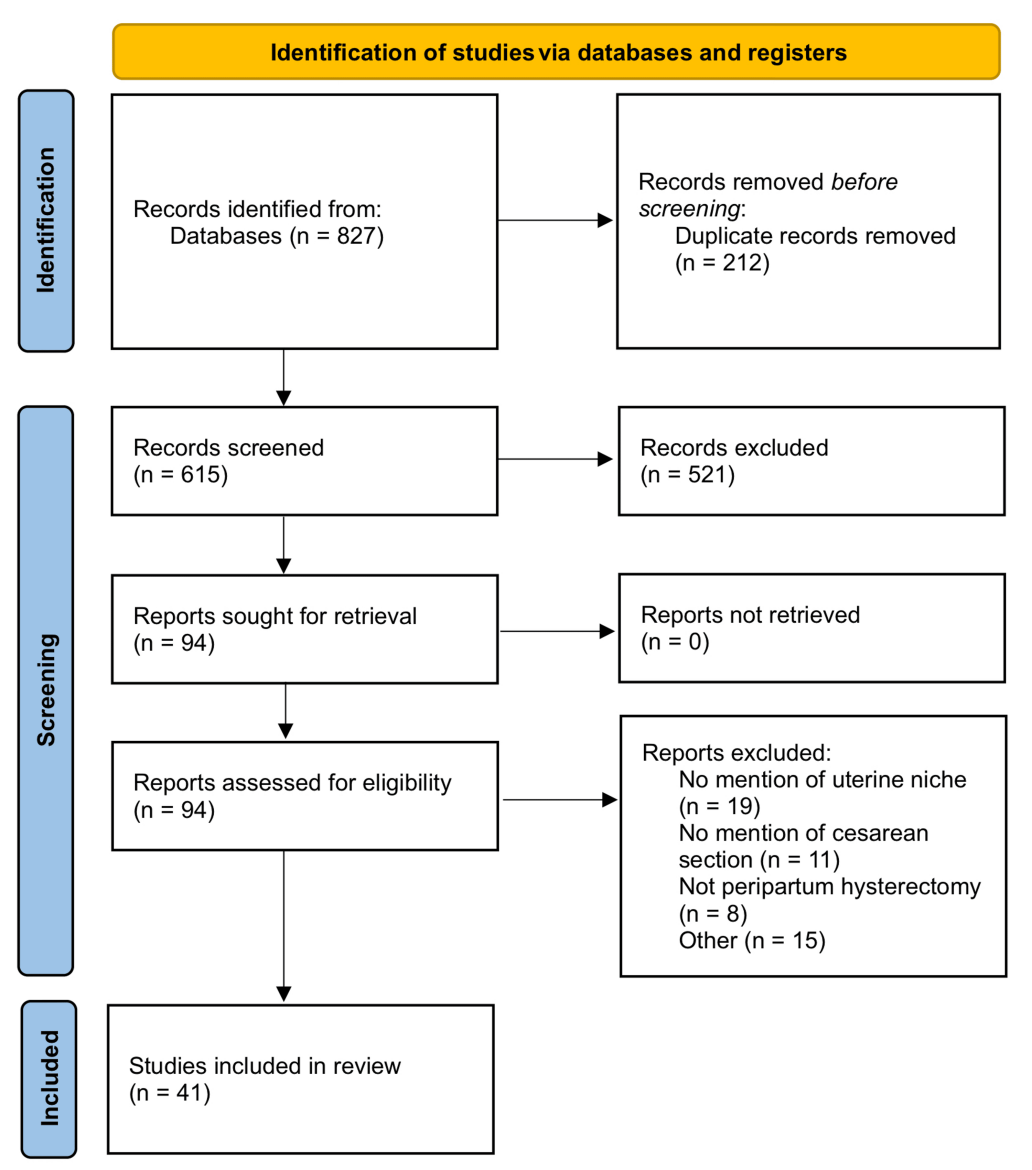
Flow diagram summarizing the literature screening process, based on the PRISMA 2020 template. PRISMA: Preferred Reporting Items for Systematic Reviews and Meta-Analyses.

The systematic review was conducted by performing an advanced literature search of PubMed and Google Scholar with a series of pertinent search terms. The utilized search terms included “uterine niche and dehiscence”, “uterine niche and prevention”, “cesarean section or hysterotomy or uterine surgery or uterine scar or niche or dehiscence or myometrium and PRP or MSC or mesenchymal stem cells”, “PRP and uterine healing”, and “uterine niche or uterine dehiscence and prevention or PRP or MSC”. The use of the aforementioned search terms produced 827 results. The initial articles were excluded if they were duplicates, not available in English, not pertinent to niche prevention, examining layers other than myometrium/endometrium, describing non-cesarean surgeries, did not examine outcomes related to the uterine niche, residual myometrial thickness (RMT), or healing ratio, or if they detailed niche interventions outside of the intra-operative or immediate pre-/post-operative period. Inclusion criteria were used to choose any peer-reviewed articles describing interventions to prevent uterine niche formation or subsequent, related complications. Human, animal, and in-vitro studies were all included if pertinent to myometrial/endometrial healing following a hysterotomy. Any time of publication was accepted, albeit it should be noted that most of the articles used were published during the past couple of decades. With the use of the selected inclusion and exclusion criteria, 41 peer-reviewed references were ultimately utilized for this systematic review. Figure 1 demonstrates the study selection flowchart.

Results

The etiology of uterine niche formation can be broadly summarized into three categories: location of hysterotomy incision, closure technique, and factors related to wound healing [7]. Aside from incidence, the severity of uterine niche is also an important factor in consideration of future obstetric complications, as residual myometrial depth has shown a strong negative predictive value for the incidence of dehiscence and rupture [6]. There is no consensus grading system to classify uterine niche severity, rather, it is based on current literature. A niche is usually defined as a myometrial indentation of more than 2 mm depth [3,7]. A large niche may be described by a depth of more than 50% or even 80% of the myometrial wall, with variations between different studies, or residual myometrial thickness less than 2.2 mm (TVUS) or 2.5 mm (SHG) [3,6,7]. An additional consideration for obstetric complications of uterine niche may be the interval between a C-section and subsequent pregnancy. For instance, the risk of uterine rupture, secondary to a niche, is the greatest with an inter-delivery interval of less than 18 months [8].

Location and Timing of Hysterotomy Incision

The location of the hysterotomy incision appears to be an important factor in niche development. In fact, very low uterine incision has been demonstrated as an independent risk factor for large uterine niches in a 2010 clinical study [9]. Later research has hypothesized that very low uterine incisions, in combination with cervical effacement, can cause the incision to be made through cervical tissue [3,10]. Indeed, incisions of C-sections performed after the onset of uterine contractions often involve the cervix [11]. As a result, mucus production from involved cervical tissue may accumulate in communicating spaces, form retention cysts, and contribute to the increased size of the uterine niche [3]. Additional studies support this theory, as high uterine niche incidence has been associated with lower station, cervical dilation greater than 5 cm, and the onset of labor > 5 hours before C-section [6,12]. In one retrospective study, half of the women with large niches had a C-section performed with advanced cervical dilatation (> 8 cm), whereas only 9% of large defects with a closed cervix [13]. Furthermore, multiple studies advise against the routine creation of a bladder flap prior to uterine incision, as it has been correlated with lower uterine incisions and a higher incidence of uterine niche formation [7,10]. Most recently, a 2022 study highlights both low uterine incision and the use of bladder flap as two preventable risk factors for isthmocele formation [10].

Uterine Closure Technique

Closure techniques have been implicated as important factors responsible for isthmocele formation; however, the outcomes are conflicting. The closure debate is mostly centered on the use of single- vs. double-layered closure, in addition to suture/knot technique and choice of material. It should be mentioned that the large-scale cesarean section surgical techniques such as CORONIS and CAESAR trials found no significant difference in immediate outcomes between single- vs. double-layer closure; however, these studies did not look at long-term uterine integrity or obstetric complications in subsequent pregnancies [14,15]. Meta-analyses of single- vs. double-layered closures generally supported a significantly greater RMT with double-layered closures, without associated differences in future dehiscence, or rupture [16-18]. Clinically, the incidence of uterine rupture in TOLAC (trial of labor after cesarean section) appears unaffected by the choice of single- or double-layered closure [18,19]. However, in terms of uterine niche formation, one meta-analysis of 15,053 women did support significant reductions in incidence with double-layer closure [17]. Increased incidence of niche formation was also correlated to the exclusion of the decidua in closure [17]. Interestingly, some recent randomized controlled trials found no significant difference in the incidence and size of cesarean scar defects between single- or double-layered suture techniques, with one Dutch study demonstrating slightly better outcomes with single-layered closure [20-23]. The Dutch randomized controlled trial (RCT) included 1,544 patients and found a 4.7% higher incidence of uterine niche formation with double-layered closure [21]. However, this outcome contrasts the majority of RCTs, where double-layered sutures achieved significantly greater RMT and healing ratio (RMT/adjacent myometrial thickness), and decreased niche incidence compared to single-layer closure [3,8,17,18,24-26]. It should be noted that it was the same Dutch team that conducted the aforementioned meta-analysis with 15,053 women, that endorsed better outcomes with double-layered closure [17]. The discrepancy in outcomes has been proposed to be attributed to double-layered closures generally producing more favorable outcomes on imaging (TVUS or SHG), but no considerable clinical effect in terms of reducing future obstetric complications [18]. In addition, the choice of thread material in closure does not appear to have any significant effect on RMT [8].

The different closure techniques have inherent benefits and risks, as double-layering and locking sutures may cause tissue strangulation and impaired wound healing but may also have better immediate approximation compared to a single-layered suture [8,13,17]. Another obvious benefit of single-layered suturing is decreased operative time by more than six minutes [7,18]. Because of the different properties of the techniques, an optimal closure protocol may utilize a combination of their benefits. In randomized controlled trials and a meta-analysis, a significantly increased RMT and healing ratio and decreased niche incidence were achieved with a double-layered suture, where the first layer was unlocked [17,25,26]. The results were not comparable when the first layer was locked [23,25]. Furthermore, the different techniques may be indicated for different clinical settings, as unlocked double-layered sutures can be very useful in preventing uterine niche (improved approximation) if tissue strangulation is avoided, whereas single-layer techniques may be optimal for thin myometrial edges (i.e., repeat cesareans) [27].

Closure technique may also exhibit different outcomes based on the size of the resultant uterine defect. For instance, a prospective cohort study found no significant difference in the overall incidence of uterine niche (> 2 mm depth) depending on single- vs. double-layered closure; however, single-layered closures were associated with five-fold increased odds of large uterine niche [28]. However, these outcomes may be difficult to interpret without a standardized definition of a “large” niche.

Most recently, a couple of randomized control trials have shown great promise with a continuously modified vertical mattress closure, known as the Babu and Magon technique. One study compared the Babu and Magon closure with a double-layer closure and found a significantly decreased risk of niche and large niche formation [29]. Comparable results have been observed when compared to single-layer closure [30], but support from larger trials is still needed to establish reliability. Nevertheless, it highlights that outcomes may be highly dependent on the skill/expertise of the surgeon in the selected closure technique. For instance, the double-layered closure has been used extensively in the UK with positive outcomes (note that most recent 2021 National Institute of Health and Care Excellence (NICE) guidelines specify no difference in outcomes based on closure technique) [31,32], whereas the single-layer technique has been associated with better outcomes in the Netherlands [21]. Ultimately, repeated use and expertise of a given technique in a specific surgical team may lead to better outcomes with that closure technique compared to other, less perfected, techniques. Table 1 summarizes uterine niche incidence/size, RMT, healing ratio, or uterine rupture as outcomes of single- vs. double-layer uterine closure as independent variables.

**Table 1:** Summary of three meta-analyses, nine randomized control trials (RCTs), and three prospective cohort studies examining uterine niche incidence/size, RMT, healing ratio, or uterine rupture as outcomes of single- vs. double-layer uterine closure as independent variables. SL (single-layer) and DL (double-layer) closure are used for conciseness. SL: single layer, DL: double layer, RMT: residual myometrial thickness, RR: relative risk, AMT: adjacent myometrium thickness.

Authors and Year	Country	Study Design	Outcomes
Vachon-Marceau et al. (2017) [8]	Canada	Multicenter cohort study (n = 1,613)	Significantly increased RMT (0.11 mm, 95% CI, 0.02-0.21) and decreased risk of uterine niche (RR: 0.32, 95% CI, 0.17-0.61) w/ DL (vs. SL) closure. No difference between types of thread.
Di Spiezio Sardo et al. (2017) [16]	International	Meta-analysis of 9 RCTs (n = 3,969)	Significantly decreased RMT (-2.19 mm, 95% CI, -2.80 to -1.57) w/ SL (vs. DL) closure. No significant difference in uterine niche and rupture.
Stegwee et al. (2018) [17]	International	Meta-analysis of 20 RCTs or prospective cohort studies (n = 15,053)	Significantly decreased RMT (-1.26 mm, 95% CI, -1.93 to -0.58) and healing ratio (RMT/AMT) (-7.74%, 95% CI, -13.31 to -2.17), and increased uterine niche incidence (RR: 1.71, 95% CI, 1.11-2.62) w/ SL (vs. DL) closure.
Qayum et al. (2021) [18]	International	Meta-analysis of 18 RCTs (n = 16,303)	Significantly reduced RMT (-1.15 mm, 95% CI, -1.69 to -0.60) w/ SL (vs. DL) closure.
Tekiner et al. (2018) [20]	Turkey	Prospective cross-sectional cohort (n = 280)	No significant difference in the size of the uterine niche w/ SL vs. DL closure.
Stegwee et al. (2021 )[21]	Netherlands	RCT (n = 2,292)	Significantly increased niche incidence (4.7%, 95% CI, 0.7-8.7%) w/ DL (vs. SL) closure.
Bamberg et al. (2017) [22]	Germany	RCT (n = 435)	No significant difference in niche incidence or size. Increased RMT with DL (vs. SL) closure (p = 0.06).
Yilmaz Baran et al. (2021) [23]	Turkey	RCT (n = 225)	No difference in uterine niche incidence w/ SL vs. DL locking closure.
Sevket et al. (2014) [24]	Turkey	RCT (n = 36)	Significantly increased RMT (9.95 vs. 7.53 mm, 99% CI) and healing ratio (0.83 vs. 0.67, 99% CI) w/ DL (vs. SL) closure.
Roberge et al. (2016) [25]	Canada	RCT (n = 73)	Significantly increased RMT (2.3 mm, 99% CI) and healing ratio (19%, 99% CI) w/ DL (1st layer non-locking) (vs. SL) closure.
Hanacek et al. (2020) [26]	Czech Republic	RCT (n = 324)	Significantly wider uterine niches (p = 0.002) and decreased RMT (p = 0.019) w/ SL closure (vs. DL). Hysterotomy performed at full cervical dilation was significantly closer to the external cervical os (p = 0.0001) (vs. minimal dilation).
Kataoka et al. (2016) [28]	Japan	Prospective cohort study (n = 267)	No difference in uterine niche incidence, but significantly increased odds of the large defect (5.59, 95% CI, 1.71-18.28) with SL (vs. DL) closure.
Tahermanesh et al. (2021) [29]	Iran	RCT (n = 72)	Significantly decreased incidence of the niche (23.5 vs. 50%, 95% CI) and large niche (2.9 vs. 23.7%, 99% CI) w/ Babu and Magon (vs. DL) closure.
Kalem et al. (2021) [30]	Turkey	RCT (n = 138)	Significantly increased RMT (p < 0.001) and decreased niche incidence (p < 0.001) w/ Babu and Magon (vs. SL) closure.
Bayraktar et al. (2022) [33]	Turkey	RCT (n = 194)	No difference in uterine niche incidence w/ locked vs. non-locking SL closure.

Adhesion Prevention

**Figure 2:**
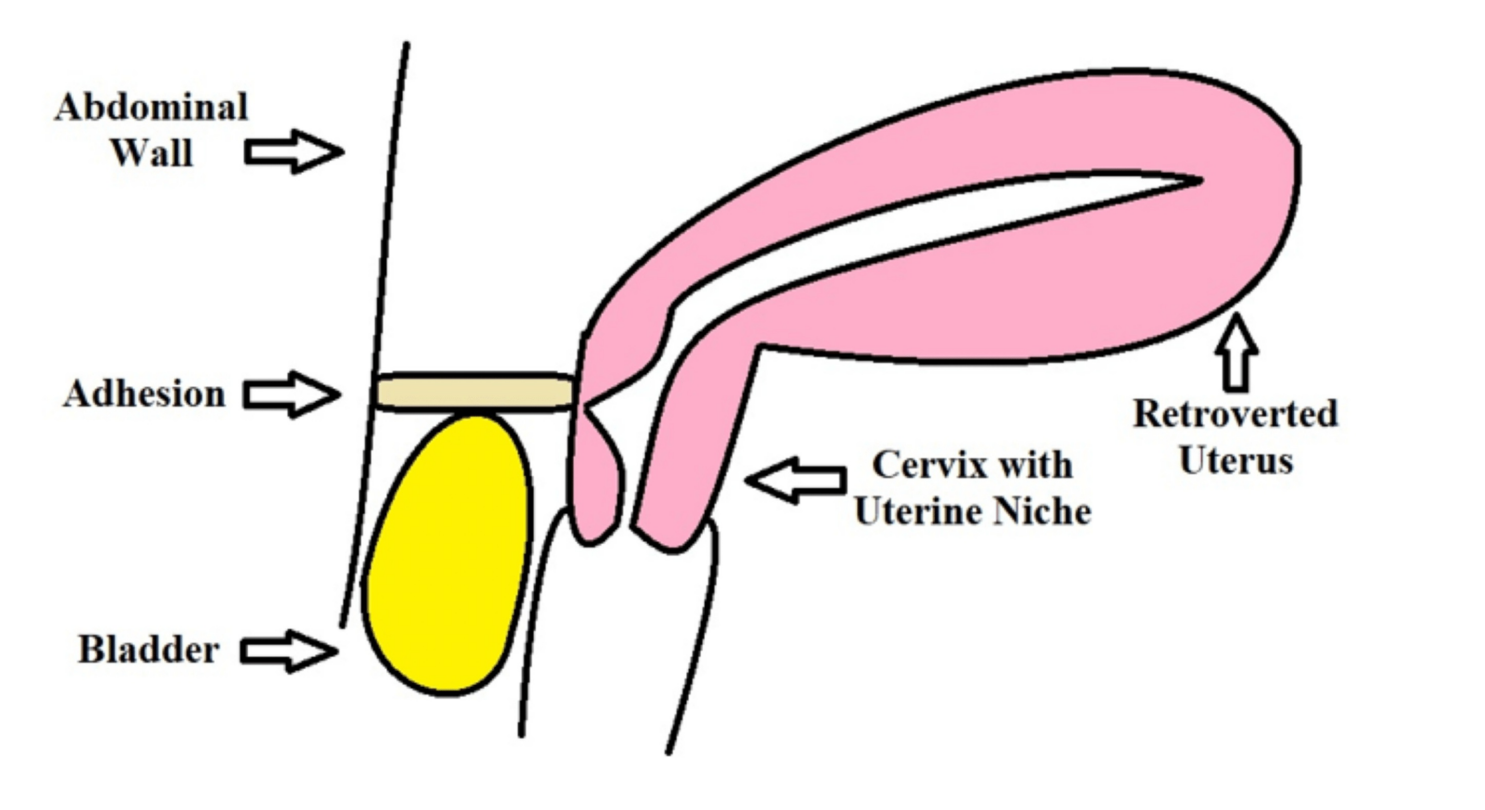
Visual illustration of a retroverted uterus experiencing retracting forces from post-operative adhesions between the abdominal wall and uterus at the apex of the niche. The figure is produced by the authors.

Some theories on uterine niche etiology suggest that adhesions in the abdominal wall may attach to the uterine scar and retract it superficially, in the opposite direction of the retracting forces in the myometrial closure (Figure 2) [7]. The result may be a suboptimal approximation and healing of the myometrium. The theory is supported by the frequent observation of adhesions attached between the uterus, at the apex of a myometrial niche, and the abdominal wall or bladder flap [7]. The vectors of the counteracting forces may be even more opposing in a retroflexed uterus, which may account for the widely recognized correlation between uterine retroflexion and uterine niche [3,6,7,13,28]. It is unclear if a retroverted uterus is a risk factor for niche formation or a consequence of niche formation [7]. A proposed step in the prevention of adhesions is to perform a peritoneal closure, which is not done frequently nowadays as suturing of the peritoneal layers may be redundant and demonstrates no improved short-term outcomes [7,27,34]. However, the current guidelines are not based on long-term outcomes [27]. Adhesion formation (and possibly uterine niche) has been observed as a long-term outcome of peritoneal non-closure in meta-analysis [7,27,34]. The use of other methods, such as adhesion barriers (i.e., sodium hyaluronate-carboxymethylcellulose), has not shown significant efficacy in preventing adhesions or uterine dehiscence in randomized trials [7,35].

Intra-operative Injections of Platelet-Rich Plasma or Mesenchymal Stem Cells

The use of platelet-rich plasma (PRP) in improving scar healing has recently been proposed. A couple of randomized trials have shown improved outcomes of redness, edema, ecchymosis, discharge, and approximation (REEDA) scale and Vancouver Scar Scale (VSS) with intra-operative PRP injections at the site of abdominal wall closure [36,37]. Perhaps more relevant is the intra-operative application of PRP and mesenchymal stem cell (MSC) as a myometrial injection. PRP and MSC have demonstrated potential in promoting endometrial regeneration and have been used accordingly for endometrial scarring (i.e., Asherman syndrome), but this regenerative potential could also benefit endometrial/myometrial healing post-hysterotomy [38-41]. A 2022 randomized controlled trial examined the intra-operative injection of PRP between the decidua and myometrium at the closure site versus a placebo [41]. Uterine niche incidence was four times greater in the placebo control group, and the intervention group also had significantly increased RMT and significantly reduced niche depth [41]. The main weaknesses of the evidence are the small sample size and that no similar studies have been performed elsewhere.

Human mesenchymal stem cells can be isolated from somatic tissues such as bone marrow, adipose, or fetal tissue and can exhibit a major role in the regulation of smooth muscle cell expression and function [40,42]. A recent animal study using human Wharton’s jelly MSC found complete restoration of full-thickness uterine injuries, a significant improvement to placebo [40]. However, in-vivo perinatal use of MSC lacks clinical evidence. MSC has demonstrated the capacity to reduce fibrosis and improve endometrial healing following repair of intrauterine adhesions in clinical settings [43]. Furthermore, there are early clinical studies that indicate the potential benefits of intra-uterine MSC injections on postpartum outcomes [44]. One study of 53 patients did show a significant reduction in postpartum inflammatory conditions, such as endometritis/myometrial infection, with an injection of 500 µl of MSC suspension in the closed myometrium, compared to placebo [45]. Although the primary outcome is not uterine niche, prevention of postpartum infection may prevent adhesion formation and indirectly protect against niche formation [45]. Another clinical trial examined intra-uterine MSC injections in postpartum women with pre-existing adhesions or uterine niches and concluded it had a safe clinical profile and exhibited improvement of RMT and niche depth reduction, albeit not significant [44]. It remains unclear if the difference would be significant with MSC administered intra-operatively, in contrast to postpartum. Conveniently, a randomized control trial of 120 patients, examining intra-operative MSC versus placebo injections with uterine niche incidence as the primary outcome, was approved in 2017, but the results have yet to be published [42].

Alpha Lipoic Acid Supplement

Alpha lipoic acid (ALA) can exhibit properties of enhanced uterine wound healing by tissue-specific upregulation of alpha-smooth muscle actin (aSMA) and vascular endothelial growth factor (VEGF), which leads to increased myofibroblast activity and angiogenesis. respectively [46]. A recent triple-blinded randomized trial of 102 women undergoing C-sections looked at the use of ALA supplements to promote optimal uterine healing [47]. ALA was administered for six weeks postpartum, as film-coated tablets with 600 mg dosing per oral (PO) bid, with half the participants receiving a placebo tablet [47]. Interestingly, results indicated a significant reduction of uterine niche incidence (60.8% vs. 80.4%), uterine niche depth (1.61 vs. 3.75 mm), and significant increases in RMT and healing ratio for the intervention group compared to placebo [47]. ALA supplementation in the postpartum period may provide a convenient, non-invasive method of uterine niche prevention if these results can be reproduced in larger clinical studies.

Discussion

The results of this literature review provided an overview of current research on the etiology, risk factors, and potential preventative measures of uterine niche formation from C-sections. Although the etiology appeared to be highly multifactorial, a few main categories of pathophysiology can be elicited: protocol of hysterotomy incision, closure technique, and factors related to wound healing [7]. In terms of hysterotomy, it is widely accepted that a very low uterine incision is a major risk factor for uterine niche formation and depth [3,7,9]. The proposed pathophysiology attributes this relationship to the formation of mucus-containing retention cysts impairing approximation, as very low incisions may involve cervical mucus-producing tissue [7,9-11]. Other factors such as increased cervical dilation, low station, long duration since onset of labor, and creation of a bladder flap have also been correlated with uterine niche, as they are likely to predispose very low hysterectomies [6,7,10,12,13]. Cervical dilatation in particular may complicate the visual identification of the transition between the cervix and uterus [7]. Although some of these factors may be difficult to manipulate during the management of ongoing labor, it is advisable to avoid very low uterine incisions through cervical tissue, especially in a patient with extensive cervical effacement, if uterine niche prevention is the goal. Furthermore, the creation of the bladder flap may introduce risk as studies indicate an increased risk of uterine niche formation [7,10]. This correlation may be indirectly attributed to a higher chance of a low hysterotomy (through cervical tissue) with bladder flap creation. However, as the bladder flap may be considered a necessary step in the prevention of bladder injury, more studies would be needed to definitively examine a relationship between bladder flap creation and uterine niche as an outcome. Ultimately, the location of the hysterotomy incision and the timing of the C-section are important considerations in the prevention of isthmocele formation. Ideally, a C-section performed before uterine contractions and advanced cervical effacement, with a relatively higher hysterotomy incision (avoiding incisions through cervical tissue), is likely to have a lower post-operative incidence or severity of uterine niche formation.

The debate on optimal uterine closure technique remains inconclusive, as multiple meta-analyses and randomized clinical trials endorse conflicting results. The debate centers around the use of single- versus double-layered suturing in uterine closure. Various sources describe no significant difference in the uterine niche as an outcome dependent on the choice of double- or single-layered suture [16,19,20,22,23]. Some clinical trials demonstrated better niche outcomes with single-layered closure [21,29]. The argument was made that single-layered suture may lead to less tissue strangulation and subsequently better healing of the uterine incision. However, most research does support optimal uterine niche outcomes with closures using double-layered, non-locking sutures, which may be supported by better wound approximation [3,8,17,18,24,25,27]. Interestingly, an ideal choice of closure technique can also vary by geographic region as the United Kingdom reports optimal outcomes with double-layered closure [27,31,32], compared to better results with single-layered closure in Dutch healthcare [21]. These variations may highlight outcomes dependent on the clinical expertise of the surgeon to a greater degree than the inherent properties of the technique. The NICE guidelines for C-sections in the UK have advised double-layered closure for numerous years (until most recently supporting the choice of either technique in 2021) [31,32], and consequently, it may be reasonable to infer that UK surgeons, trained in double-layered closure, may achieve better outcomes with their most perfected technique. Inversely, proficiency with the single-layer closure in the Netherlands may have led to better outcomes in that healthcare system [21]. Future studies should examine RMT, healing ratio, and uterine niche incidence as an outcome dependent on the surgeon’s level of experience with the selected closure technique. Nevertheless, most of the cited research endorsed reliable utilization of double-layered, with deep layer non-locking, sutures in uterine closures with the goal of preventing uterine niche formation and severity [3,8,17,24,25,27]. Single-layered closure was proposed to be highly useful in the approximation of thin endometrial edges [27]. Lastly, the continuous vertical mattress closure (Babu and Magon technique) has shown significantly improved outcomes, compared to both single- and double-layered closures, in recent randomized control trials, but larger studies are needed to reach a conclusion on efficacy [29,30]. The optimal choice of closure technique should be made based on the individual expertise of the surgeon, as all techniques have strengths and weaknesses.

Recurrent clinical observations described the presence of intra-abdominal adhesions attached to the uterus at the apex of a uterine niche [7]. It was theorized that the post-operative formation of adhesions may impose superficially retracting forces on the uterine scar, in the opposing direction of the uterine closure, thus impairing approximation, healing, and increasing the risk of a large uterine niche [7]. The opposition of force vectors may be even more prominent in a retroverted uterus, which could account for the strong correlation between uterine niche and retroverted uteri [3,6,7,13,28]. A proposed intervention to prevent this pathophysiology was the closure of the peritoneum. Non-closure of the peritoneum is generally the mainstay protocol. However, multiple meta-analyses highlight a significant correlation between adhesion formation and non-closure of the peritoneum [7,27,34]. Although research has demonstrated no significant difference in post-operative outcomes between closure and non-closure, these studies looked at short-term complications and may not account for the possible prevention of future uterine niches with peritoneal closure, which may take around six months to develop [27]. Despite the correlation between adhesions and peritoneal non-closure, randomized clinical trials would be needed to examine the effect of peritoneal non-closure on niche development. The current studies supporting closure are few and have yet to examine niche formation or future obstetric outcomes as primary or secondary outcomes. Furthermore, there is minimal research on the etiology to explain the correlation between adhesions and uterine niches. Nevertheless, peritoneal closure could be an intervention to prevent uterine niche and subsequent long-term complications. The use of adhesion barriers did not exhibit any improved outcomes in terms of adhesion formation or uterine dehiscence. Other methods of adhesion prevention should be considered for future research.

Aside from procedural interventions, recent research also suggests potential adjunct applications of PRP and MSC to reduce the incidence and severity of uterine niches [41-45]. PRP and MSC were examined as intra-operative injections between the decidua and myometrium at the uterine closure site [41,42,45]. PRP, in particular, has demonstrated potential in endometrial regeneration and management of Asherman’s syndrome, in addition to improved superficial scar healing from C-sections [36,37]. It raised the question of whether PRP may also improve myometrial healing and niche prevention following hysterotomy. As of 2022, there is only one randomized controlled trial examining uterine niche as an outcome with PRP injection versus placebo injection as the independent variable [41]. Results from the trial showed that PRP injections were correlated with a four times lower incidence of niche, significantly reduced niche depth, and significantly increased RMT when compared to placebo [41]. Although the sample size was small, the findings were encouraging and indicated a potential use as an adjunct treatment, given additional evidence from large clinical trials. MSC has also shown some potential, likely due to its regulation of smooth muscle cell expression and function [40,42]. Animal studies have supported the immense myometrial regenerative capacity of MSC [40]; however, these findings have not been replicated in the early and few clinical studies. Early clinical trials have demonstrated a good safety profile, limited myometrial regeneration, and reduction of post-operative inflammatory conditions in C-sections with myometrial MSC injections [43-45]. The reduction of inflammatory complications may be relevant as the reduction of adhesion formation secondary to inflammation may also prevent future niche formation. Direct reduction of uterine niche severity was seen in one clinical trial with MSC injections; however, the injections were performed on patients with pre-existing niches and the results were not significant [44]. A randomized clinical trial of uterine niche as a dependent variable of intra-operative, myometrial MSC injections was approved in 2017, but any results have yet to be published [42]. As with PRP, clinical research on MSC injections as an adjunct in niche prevention is encouraging but minimal. Another issue with these interventions is the lack of standardized definitions for PRP and MSC. As long as there is no clear definition for the components and concentrations of these injections, there can be no conclusions made about their clinical significance.

Lastly, alpha lipoic acid (ALA) as an oral postpartum supplement following a C-section showed significant clinical benefit on niche outcomes in one recent triple-blinded randomized controlled trial [47]. Initiation of an oral, bidaily, 600 mg ALA capsule in the immediate postoperative period after a C-section, for a six-week duration, was the intervention compared to placebo [47]. As intervention showed a significant reduction of niche incidence and depth, it may be a relevant topic to examine in future clinical research. Ideally, an ALA supplement could be a simple, non-invasive, low-risk adjunct to decrease the risk of uterine niche.

Ultimately, the optimization of protocol to facilitate myometrial healing during cesarean sections could decrease the incidence and severity of uterine niches. As evidence, research is ongoing, and multiple procedural components lack consensus. Moving forward, uterine niche and associated complications as a primary outcome of cesarean protocol need to remain the focus of research. As C-section rates continue to rise globally, uterine niche formation and correlated obstetric complications are likely to become increasingly burdensome for healthcare.

## Conclusions

The following intra-operative protocols may yield optimal cesarean outcomes in terms of niche prevention: avoiding a very low uterine incision, timing of hysterotomy prior to extensive cervical effacement, avoiding the creation of a bladder flap, utilization of the uterine closure technique most familiar to the surgeon (general recommendation is double-layered suture with the deep layer non-locking), and possibly peritoneal closure. In addition, intra-operative injections of PRP and/or MSC between the decidua and myometrium at the closure site and postpartum alpha lipoic acid supplement have shown promise but are not currently recommended due to the lack of evidence. Note that this single review cannot provide a comprehensive perspective of all the possible clinical approaches and that certain theories are still under debate. These suggestions should not replace clinical experience and should be considered tentative. The conclusions may be applicable in the appropriate setting where optimal future obstetric outcomes and minimal post-operative gynecologic complications are prioritized.
