# Correction: Intra-operative Guidelines for the Prevention of Uterine Niche Formation in Cesarean Sections: A Review

**DOI:** 10.7759/cureus.c261

**Published:** 2025-08-26

**Authors:** Sean Backer, Deepesh Khanna, Sonia Sadr, Ali Khatibi

**Affiliations:** 1 Osteopathic Medicine, Nova Southeastern University Dr. Kiran C. Patel College of Osteopathic Medicine, Tampa, USA; 2 Foundational Sciences, Nova Southeastern University Dr. Kiran C. Patel College of Osteopathic Medicine, Clearwater, USA; 3 Foundational Sciences, Nova Southeastern University Dr. Kiran C. Patel College of Osteopathic Medicine, Fort Lauderdale, USA; 4 Obstetrics and Gynaecology, Sahlgrenska University Hospital, Gothenburg, SWE

This article has been corrected to fix the following typo in the PRISMA diagram: "Records excluded" has been corrected from 571 to 521.

